# Acceptance of Selective Laser Trabeculoplasty as a First-Line Treatment for Primary Open-Angle Glaucoma in Saudi Arabia

**DOI:** 10.7759/cureus.52360

**Published:** 2024-01-16

**Authors:** Manal Alwazae, Atheer Alhumud, Shrouq Alsarhan, Leyla Ali Aljasim

**Affiliations:** 1 Ophthalmology, King Khaled Eye Specialist Hospital, Riyadh, SAU; 2 General Medicine, King Saud Medical City, Riyadh, SAU; 3 General Medicine, Princess Nourah Bint Abdulrahman University, Riyadh, SAU; 4 Glaucoma, King Khaled Eye Specialist Hospital, Riyadh, SAU

**Keywords:** saudia arabia, primary open angle glaucoma, practice pattern, slt, selective laser trabeculoplasty

## Abstract

Purpose: Glaucoma is the second leading cause of blindness worldwide. Early detection and timely treatment are crucial to reducing disease progression. Selective laser trabeculoplasty (SLT) has proven efficacious as a primary treatment for primary open-angle glaucoma. This study aims to evaluate the acceptance among Saudi ophthalmologists of using SLT as a primary treatment for glaucoma.

Methods: This cross-sectional study enrolled 128 ophthalmologists practicing in Saudi Arabia. Data collection was conducted using a structured online questionnaire, which evaluated sociodemographic data, current glaucoma practice, the technology acceptance model (TAM), and potential barriers to incorporating SLT as the primary treatment for glaucoma.

Results: The mean age of the participants was 40 ± 9.6 years, with 65.6% being male. Almost one-third were glaucoma specialists, and 89% followed the American Academy of Ophthalmology recommendations for managing glaucoma patients. The majority (96.1%) used medical treatment as the initial therapy, 72.7% agreed that SLT is safe, and 59.4% agreed that it rapidly controls intraocular pressure. Nearly half of the participants were willing to use SLT as the primary treatment, yet only 42.2% considered themselves experienced enough to do so. The most reported barriers were inadequate training (47.7%), non-availability of SLT equipment (41.4%), and low efficacy as reported by 27.3% of participants.

Conclusion: Despite the good overall acceptance of SLT as a first-line treatment for glaucoma, most participants still preferred medical therapy as the primary treatment. To overcome the barriers to incorporating SLT, Saudi ophthalmologists require more training and access to equipment to effectively implement this modality in their practices.

## Introduction

Glaucoma is a progressive disease that gradually damages the optic disc, leading to surreptitious visual loss [1]. It is the second leading cause of irreversible blindness worldwide [2]. In Saudi Arabia, glaucoma accounts for 3% of blindness in individuals over 40 years of age [3]. Therefore, early diagnosis and appropriate treatment are crucial in mitigating the progression of this disease. There are various treatment modalities for glaucoma, most of which target intraocular pressure (IOP). These treatments include medical therapy, laser trabeculoplasty, and surgery. However, the selection of treatment remains challenging and must be tailored to patient characteristics and the risk profile, including the type of glaucoma, optic disc status, IOP, patient literacy, compliance, and affordability [4].

The application of evidence-based guidelines for managing glaucoma is essential to establish effective and comprehensive ophthalmic care. Surprisingly, studies have reported suboptimal adherence among ophthalmologists to the recommended management protocols, such as those outlined by the American Academy of Ophthalmology (AAO) [5-10]. The AAO has produced a management guideline called the Preferred Practice Pattern that strongly recommends decreasing IOP by 20-30% through medical or surgical intervention as initial treatment [11]. Although most ophthalmologists prefer to start treating glaucoma with topical medications, patient compliance remains a challenge [4].

Recent studies have indicated that selective laser trabeculoplasty (SLT) can be used as a primary treatment for glaucoma. For example, the Laser in Glaucoma and Ocular Hypertension Trial (LiGHT) reported that SLT is equally effective as topical medications [12]. Additionally, SLT reduces the number of topical glaucoma medications needed to control the disease for several years [13]. SLT is safe and can be repeated effectively without damaging the trabecular meshwork [13,14]. Furthermore, it is cost-effective because it significantly reduces the overall expense of care compared to medications and more invasive surgeries [14].

There is a relative paucity of data from Saudi Arabia on the current practices for managing glaucoma and the acceptance of ophthalmologists for using SLT as a primary treatment for glaucoma. The objective of this study was to evaluate the acceptance among Saudi ophthalmologists of using SLT as a first-line (primary or initial) treatment for glaucoma.

## Materials and methods

Study design, setting, and participants

This cross-sectional study was conducted from February to July 2020. It was approved by the Institutional Review Board of King Khaled Eye Specialist Hospital (approval number 2042-P).

The study sample comprised 128 ophthalmologists working in Saudi Arabia, including professionals from all ophthalmic subspecialties and senior residents who had completed the glaucoma rotation in their program. The participants were requested to respond to a questionnaire regarding their current glaucoma management patterns.

The sample size was calculated based on the assumption that 30 ± 10 ophthalmologists accepted the use of selective laser trabeculoplasty (SLT) as the primary treatment for glaucoma. G-Power software was utilized for calculating the minimal sample size. With a 95% level of significance (α=0.05) and a power of 80% (β=0.2), it was determined that at least 126 ophthalmologists needed to be enrolled in the study.

Data collection

A structured questionnaire was employed for data collection. Its validation and wording were tailored to the objectives of this study. An anonymous survey was created using the online services provided by surveymonkey.com and distributed via email link. The questionnaire consisted of four main parts: sociodemographic data, current glaucoma practice, the Technology Acceptance Model (TAM) questionnaire, and barriers to using SLT as a first-line treatment for glaucoma.

Sociodemographic data collected included age, gender, ophthalmology subspecialty, training program, and practice setting. The second part of the questionnaire focused on glaucoma management in terms of the initial choice of therapy and the frequency of using SLT as a first-line treatment. The acceptance and satisfaction of using SLT as a first-line treatment were assessed using the TAM [15], which is divided into five sections: Perceived Usefulness (four statements), Perceived Ease of Use (three statements), Individual Behavioral Intention to SLT Adoption and Use (four statements), Actual SLT Used by Physicians (four statements), and Physician Satisfaction with SLT (four statements). Each section is graded on a 3-point Likert scale: 1 (disagree), 2 (neutral), and 3 (agree).

The final part of the questionnaire addressed potential obstacles to using SLT as the primary treatment, with a list of factors where participants could choose multiple responses. The list included patient refusal, non-availability, poor efficacy, and cost, among others.

Two independent experts, a glaucoma specialist and an epidemiologist, evaluated the content and face validity of the modified version of the TAM questionnaire. The internal consistency of the questionnaire was high, with a Cronbach's alpha of 0.81 for 19 items.

Statistical analysis was conducted using SPSS version 23 (IBM Corp., Armonk, NY, USA). Descriptive statistics are reported as proportions, means, and standard deviations. Quantitative data were analyzed with the t-test, and the chi-square test was used to assess the association of qualitative variables. A p-value <0.05 was considered statistically significant.

## Results

Table 1 presents the sociodemographics and other characteristics of the study sample. The majority, 84 (65.6%), of the participants were male. The mean age was 40 ± 9.6 years. Almost one-third of the respondents were glaucoma specialists, and the majority, 87 (68%), were Saudi board-certified. Most of the participants, 62 (48.4%), practiced ophthalmology at tertiary care centers and specialized centers.

**Table 1:** Sociodemographic data of ophthalmologists who participated in the questionnaire on the management of glaucoma. *Other boards include Australian, Arab, British, and Tunisian boards. SD denotes standard deviation.

Characteristics	Frequency (%)
Age in years, mean ± SD (years)	40 ± 9.5
Gender	
Male	84 (65.6)
Female	44 (34.4)
Ophthalmology subspecialty	
Glaucoma	36 (28.1)
Anterior segment	24 (18.8)
Retina	20 (15.6)
Pediatrics	16 (12.5)
Oculoplastic	5 (3.9)
General ophthalmology	18 (14.1)
Specialist and senior resident (PGY-4)	9 (7.1)
Ophthalmology board	
Saudi	87 (68)
Pakistan Diploma	8 (6.2)
Egyptian	6 (4.7)
Syrian	4 (3.1)
European	3 (2.3)
Jordanian	2 (1.6)
Canadian	2 (1.6)
American	2 (1.6)
Other*	14 (10.9)
Practice setting	
Tertiary care and specialized center	62 (48.4)
Ministry of Health Hospital	34 (26.6)
Private clinic	17 (13.3)
Teaching hospital	15 (11.7)

Most of the ophthalmologists, 123 (96.1%), used topical eye drops as the first-line therapy for glaucoma, and only 4 (3.1%) considered SLT as an initial treatment. Almost half of the glaucoma specialists reported using SLT sometimes as the initial therapy for glaucoma (Table 2).

**Table 2:** Current practice of glaucoma management (n=128). SLT denotes selective laser trabeculoplasty.

Treatment approach	Glaucoma specialists	Non-glaucoma specialists	Total
Current practice of primary management for glaucoma			
Topical eye drops	35 (97.2%)	86 (95.6%)	123 (96.1%)
Selective laser trabeculoplasty	1 (2.8%)	3 (3.3%)	4 (3.1%)
Surgery	0 (0%)	1 (1.1%)	1 (0.8%)
Frequency of using SLT for the primary management of glaucoma			
Never	15 (41.7%)	60 (66.7%)	76 (59.4%)
Sometimes	17 (47.2%)	24 (26.7%)	41 (32%)
Often	2 (5.6%)	5 (5.6%)	8 (6.2%)
Always	2 (5.6%)	1 (1.1%)	3 (2.3%)

Table 3 presents the perceived barriers to using SLT as a primary treatment for glaucoma. Among glaucoma specialists, the most common barriers were low efficacy, reported by 15 (41.7%), inapplicability by 12 (33.3%), non-availability by 10 (27.8%), and inadequate training by 9 (25%).

**Table 3:** Perceived barriers to using selective trabeculoplasty as an initial treatment for glaucoma. *Other includes beginning with medical treatment first, type of glaucoma, and not a glaucoma specialist. SLT denotes selective laser trabeculoplasty.

Barrier		Glaucoma specialists	Non-glaucoma specialists	Total
Patients' age	Yes	9(25%)	22(23.9%)	31(24.2%)
	No	27(75%)	70(76.1%)	97(75%)
Patient refusal	Yes	9(25%)	17(18.5%)	26(20.3%)
	No	27(75%)	75(81%)	102(79.7%)
Low efficacy	Yes	15(41.7%)	20(21.7%)	35(27.3%)
	No	21(58%)	72(78.3%)	93(72.7%)
Inapplicability	Yes	12(33.3%)	18(19.6%)	30(23.4%)
	No	24(66.7%)	74(80.4%)	98(76.6%)
Low safety profile	Yes	1(2.8%)	5(5.4%)	6(4.7%)
	No	35(97.2%)	87(94.6%)	122(95.3%)
Non-availability	Yes	10(27.8%)	43(46.7%)	53(41.4%)
	No	26(72.2%)	49(53.3%)	75(58.6%)
Low tolerance threshold	Yes	6(16.7%)	6(6.5%)	12(9.4%)
	No	30(83%)	86(93.5%)	116(90.6%)
Cost of SLT	Yes	2(5.6%)	13(14.1%)	15(11.7%)
	No	34(94.4%)	79(85.9%)	113(88.3%)
Complications rate of SLT	Yes	2(5.6%)	4(4.3%)	6(4.7%)
	No	34(94.4%)	88(95.7%)	122(95.3%)
Inadequate training	Yes	9(25%)	52(56.5%)	61(47.7%)
	No	27(75%)	40(43.5%)	67(52.3%)
Other*	Yes	3(8.3%)	14(15.2%)	17(13.3%)
	No	33(91.7)	78(84.8%)	111(86.7%)

Table 4 presents the TAM for using SLT as a primary treatment for glaucoma. Approximately half of the glaucoma specialists agreed that using SLT improves the effectiveness of lowering IOP when it is used as a first-line treatment for glaucoma. Only 23 (63.9%) believed SLT enables them to accomplish an IOP goal more quickly. Only 2 (5.6%) of the glaucoma specialists believe performing SLT is often frustrating, and 13 (36.1%) disagreed with the statement that SLT does not require several training courses to use it effectively. Among the glaucoma specialists, only 12 (33.3%) agreed that they gained rich and diverse experiences with SLT, and 17 (47.2%) stated that they are willing to use SLT as a primary treatment for glaucoma. Half of the glaucoma specialists agreed that using SLT is beneficial in their practice, and 21 (58.3%) stated that using SLT enables them to provide a suitable option to eliminate adherence issues. The overall satisfaction with using SLT was mixed, with 15 (41.7%) of the glaucoma specialists surveyed feeling comfortable using SLT and over one-third expressing satisfaction with SLT.

**Table 4:** Technology Acceptance Model (TAM) for using selective trabeculoplasty as primary treatment for glaucoma. SLT denotes selective trabeculoplasty; IOP denotes intraocular pressure.

Attributes of selective trabeculoplasty (perceived usefulness)			
Using SLT improves the effectiveness of lowering IOP as a first-line treatment for glaucoma	Glaucoma specialists	Non-glaucoma specialists	Total
Agree	16(44.4%)	31(33.7%)	47(36.7%)
Neutral	13(36.1%)	39(42.4%)	52(40.6%)
Disagree	7(19.4%)	22(23.9%)	29(22.7%)
Using SLT gives greater control of IOP	Glaucoma specialists	Non-glaucoma specialists	Total
Agree	14(38.9%)	25(27.2%)	39(30%)
Neutral	14(38.9%)	57(62%)	71(55.5%)
Disagree	8(22.2%)	10(10.9%)	18(14.1%)
SLT enables me to accomplish IOP goals more quickly	Glaucoma specialists	Non-glaucoma specialists	Total
Agree	23(63.9%)	53(57.6%)	76(59.4%)
Neutral	11(30.6%)	36(39.1%)	47(36.7%)
Disagree	2(5.6%)	3(3.3%)	5(3.9%)
SLT helps provide a safe option in the treatment plan	Glaucoma specialists	Non-glaucoma specialists	Total
Agree	28(77.8%)	65(70.7%)	93(72.7%)
Neutral	7(19.4%)	27(29.3%)	34(26.6%)
Disagree	1(2.8%)	0(0%)	1(0.8%)
Attributes of selective trabeculoplasty (perceived ease of use)			
Performing SLT is often frustrating	Glaucoma specialists	Non-glaucoma specialists	Total
Agree	2(5.6%)	17(18.5)	19(14.8%)
Neutral	18(50%)	42(45.7%)	60(46.9%)
Disagree	16(44.4%)	33(35.9%)	49(38.3%)
SLT does not require several trainings to use it effectively	Glaucoma specialists	Non-glaucoma specialists	Total
Agree	7(19.4%)	29(31.5%)	36(28.1%)
Neutral	16(44.4%)	38(41%)	54(42.2%)
Disagree	13(36.1%)	25(27.2%)	38(29.7%)
SLT is compatible with the existing clinical workflow	Glaucoma specialists	Non-glaucoma specialists	Total
Agree	13(36.1%)	11(12%)	24(18.8%)
Neutral	9(25%)	46(50%)	55(43%)
Disagree	14(38.9%)	35(38%)	49(38.3%)
Individual behavioral intention to SLT adoption and use			
I am willing to use SLT as a first-line treatment for glaucoma	Glaucoma specialists	Non-glaucoma specialists	Total
Agree	17(47.2%)	46(50%)	63(49.2%)
Neutral	14(38.9%)	36(39.1%)	50(39.1%)
Disagree	5(13.9%)	10(10.9%)	15(11.7%)
SLT positively supports the treatment plan of my patients	Glaucoma specialists	Non-glaucoma specialists	Total
Agree	18(50%)	47(51.1%)	65(50.8%)
Neutral	14(38.9%)	41(44.6%)	55(43%)
Disagree	4(11.1%)	4(4.3%)	8(6.3%)
SLT provides me with a more efficient care service	Glaucoma specialists	Non-glaucoma specialists	Total
Agree	16(44.4%)	44(47.8%)	60(46.9%)
Neutral	14(38.9%)	47(51.1%)	61(47.7%)
Disagree	6(16.7%)	1(1.1%)	7(5.5%)
I have gained rich and diverse experiences in the SLT procedure	Glaucoma specialists	Non-glaucoma specialists	Total
Agree	12(33.3%)	42(45.7%)	54(42.2%)
Neutral	12(33.3%)	42(45.7%)	54(42.2%)
Disagree	12(33.3%)	8(8.7%)	20(15.6%)
Clinical use of selective trabeculoplasty by ophthalmologists			
SLT is beneficial in my practice	Glaucoma specialists	Non-glaucoma specialists	Total
Agree	17(47.2%)	34(37%)	51(39.8%)
Neutral	13(36.1%)	43(46.7%)	56(43.8%)
Disagree	6(16.7%)	15(16.3%)	21(16.4%)
Using SLT enables me to provide a suitable option for patients who seldom come to the hospital	Glaucoma specialists	Non-glaucoma specialists	Total
Agree	21(58.3%)	43(46.7%)	64(50%)
Neutral	10(27.8%)	43(46.7%)	53(41.4%)
Disagree	5(13.9%)	6(6.5%)	11(8.6%)
SLT helps to avoid the risk of invasive surgeries	Glaucoma specialists	Non-glaucoma specialists	Total
Agree	18(50%)	63(68.5%)	81(63.3%)
Neutral	15(41.7%)	27(29.3%)	42(32.8%)
Disagree	3(8.3%)	2(2.2%)	5(3.9%)
SLT helps to treat more patients with fewer complication rate	Glaucoma specialists	Non-glaucoma specialists	Total
Agree	21(58.3%)	60(65%)	81(63.3%)
Neutral	12(33.3%)	28(30.4%)	40(31.3%)
Disagree	3(8.3%)	4(4.3%)	7(5.5%)
Ophthalmologist satisfaction with selective trabeculoplasty			
I feel comfortable using SLT	Glaucoma specialists	Non-glaucoma specialists	Total
Agree	15(41.7%)	22(23.9%)	37(28.9%)
Neutral	12(33.3%)	49(53.3%)	61(47.7%)
Disagree	9(25%)	21(22.8%)	30(23.4%)
I feel at ease in adding SLT to my existing clinical workflows	Glaucoma specialists	Non-glaucoma specialists	Total
Agree	18(50%)	39(42.4%)	57(44.5%)
Neutral	9(25%)	40(43.5%)	49(38.3%)
Disagree	9(25%)	13(14.1%)	22(17.2%)
SLT saves money in comparison to topical medication and surgeries	Glaucoma specialists	Non-glaucoma specialists	Total
Agree	21(58.3%)	51(55.4%)	72(56.3%)
Neutral	11(30.6%)	37(40.2%)	48(37.5%)
Disagree	4(11.1%)	4(4.3%)	8(6.3%)
Overall, I am satisfied with SLT outcomes	Glaucoma specialists	Non-glaucoma specialists	Total
Agree	13 (36.1%)	29(31.5)	42(32.8%)
Neutral	18(50%)	58(63%)	76(59.4%)
Disagree	5(13.9%)	5(5.4%)	10(7.8%)

## Discussion

SLT facilitates aqueous flow through the trabecular meshwork. It is an outpatient procedure performed under topical anesthesia and is known for its excellent safety profile [16]. SLT is recommended by the AAO, European Glaucoma Society (EGS), and World Glaucoma Association (WGA) as the first-line treatment for glaucoma. This is because it helps stabilize IOP for years, reduces the number and side effects of topical medications, and eliminates patient non-compliance [12,17]. The current study evaluated the acceptance among ophthalmologists in Saudi Arabia of using SLT as the primary treatment for glaucoma.

Ninety-six percent of the ophthalmologists in this study used topical ophthalmic medications as the primary therapy for glaucoma, with only 3% using SLT as the first-line treatment. However, half of the respondents had a positive attitude towards SLT and were willing to use it as a first-line treatment. Similarly, studies by the American Glaucoma Society and Latin American glaucoma specialists report minimal usage of SLT as the initial treatment by ophthalmologists [18]. This observation may be due to inadequate training being the most common barrier to using SLT as the primary treatment for glaucoma, as identified in this study. Additionally, the non-availability of SLT in some hospitals could influence its low utility in practice.

The study also assessed the perceived usefulness of SLT, finding that 58.3% of glaucoma specialists agreed it could help eliminate patient compliance issues. This aligns with findings from many studies, including Kadasi LM et al., which reported that decreasing the burden of compliance by using SLT could be a strong factor supporting its use as a primary treatment for a lifelong disease like glaucoma [13].

Among glaucoma specialists in this study, 41.7% perceived the efficacy profile of SLT as a barrier to its implementation; this might relate to inadequate training and lack of availability, hindering physicians from experiencing firsthand the results of SLT. The LiGHT study reported that using SLT as the primary therapy resulted in better IOP control over three years and provided a constant effect on the trabecular meshwork, stabilizing diurnal IOP fluctuation [12]. A case-control study from Saudi Arabia reported that 79.6% of patients with primary open-angle glaucoma (POAG) treated with SLT experienced a 20% reduction in IOP from baseline or stopped using glaucoma medications [19]. These results are comparable to another clinical trial that used SLT as the primary therapy in POAG, maintaining 70% of patients free of topical medication for 30 months [20].

In the current study, 77.8% of glaucoma specialists and 70.7% of non-glaucoma specialists believed that SLT was a safe option and had a low complication rate. This supports previous evidence from Gazzard et al. and Kadasi et al. who reported that complications after SLT are mostly self-limiting, with no long-term effects on vision [12,13]. They also found that 5.8% of patients on topical therapy deteriorated, in contrast to only 3.8% of those in the SLT group [12]. Furthermore, no patients who were treated with SLT required surgical intervention over 36 months compared to 1.8% of patients in the topical medication group who required glaucoma surgery [12]. Even rates of cataract surgery after SLT were much lower in comparison to patients on topical therapy [12].

In the current study, 11.7% of participants perceived cost as an obstacle, and only half believed that SLT could help reduce expenses. This is because many patients prefer private hospital services to governmental hospitals where the procedure might be priced higher, leading them to opt for medical therapy as the cheaper option. However, a Canadian study reported that the savings from SLT over a three-drug therapy used for six years is $3,367 [21]. This indicates that medication costs build up over time as opposed to SLT, where payment is upfront. Hence, to reduce the future expense of medications, earlier IOP control by SLT was advised.

## Conclusions

In conclusion, despite the good overall acceptance of SLT as a first-line treatment for glaucoma, the majority of ophthalmologists in Saudi Arabia are still using medical therapy as the primary treatment. Inadequate training was considered the biggest barrier to adopting SLT as the initial therapy. Further training is required to reduce the low adoption rate and for effective implementation in routine ophthalmic practice.
